# The Role of Equestrian Professionals in Saddle Fit for Horses and Riders in the United Kingdom

**DOI:** 10.3390/ani14172495

**Published:** 2024-08-28

**Authors:** Russell MacKechnie-Guire, Jane M. Williams, Diana Fisher, Kathryn Nankervis

**Affiliations:** 1 Equine Department, Hartpury University, Gloucester GL19 3BE, UK; jane.williams@hartpury.ac.uk (J.M.W.); kathryn.nankervis@hartpury.ac.uk (K.N.); 2 Woolcroft Equine Services, May Lane, Wisbech PE13 5BU, UK; ceo@mastersaddlers.co.uk

**Keywords:** saddle fitter, therapists, coaches, communication, multi-disciplinary team

## Abstract

**Simple Summary:**

Horse owners have a responsibility to safeguard their horse’s welfare and are often assisted in this role by various equestrian professionals forming a multi-disciplinary team (MDT). Saddle fit is a complex issue, and the advice given to horse owners by equestrian professionals on matters associated with saddle fit can be influential in achieving optimal saddle fit. This study investigated how equestrian professionals assess, manage, and act upon saddle fit. Participants (*n* = 483) completed an online questionnaire, which was split into three major sections: (1) participant demographics; (2) saddle fit for the horse; and (3) saddle fit for the rider. Differences in experience and practice between professional groups were analysed. From the UK, 184 saddle fitters, 77 therapists, and 116 coaches completed the survey (*n* = 377). Significant differences existed between saddle fitters, therapists, and coaches with respect to the number of years qualified, the number of horses seen per week, the frequency with which they ask clients when they last had their saddle fitted, how they themselves assess saddle fit, and the most frequent saddle fit issues encountered. The findings provide insight into MDT working and the interactions of equestrian professionals on matters associated with saddle fit.

**Abstract:**

The horse owner and the multi-disciplinary team (MDT) are responsible for safeguarding horse welfare by ensuring the equipment being used is correctly fitted. The aim of this study was to investigate how saddle fit is assessed, managed, and acted upon by equestrian professionals. Participants (*n* = 483) completed an online questionnaire, which was split into three major sections: (1) participant demographics; (2) saddle fit for the horse; and (3) saddle fit for the rider. Descriptive statistics, Kruskal–Wallis, and univariable and multivariable analyses were performed (*p* < 0.05). Inductive content analysis identified themes from open-question responses. From the UK responses (*n* = 377), 184 saddle fitters, 77 therapists, and 116 coaches completed the survey. Compared to coaches, saddle fitters and therapists asked more frequently when their clients last had their saddle fitted (*p* = 0.0004). Saddle fitters typically assessed the saddle statically and dynamically vs. therapists, where it was dependent on the circumstances of the assessment (*p* = 0.0004). Saddle fitters experienced the saddle being out of balance more than therapists (*p* = 0.032) and made more alterations to the saddle than therapists and coaches (*p* = 0.0004). This study highlights opportunities for professions within the MDT to better support each other and horseowners to achieve improvements in overall fit for horses and riders.

## 1. Introduction

There is a growing body of evidence of the influence that equipment, including a saddle, can have on the horse’s welfare, physiological responses, and performance during ridden exercise [1,2,3,4,5,6,7,8,9,10,11]. Despite evidence of the effect that sub-optimal saddle fit can have on the horse [1,2,3,4,5,6,7,8,9,10,11,12,13,14,15], several studies report a high prevalence of incorrectly fitted saddles. In a study that included 506 horse-rider combinations in the United Kingdom (U.K.), a third of saddles were reported to be out of balance when saddle fit was judged statistically by two veterinarians [14]. Similar results were reported in a riding horse population in Switzerland [16], where 90% of saddles (*n* = 196) were considered sub-optimally fitted by two experienced veterinarians, even though 95% of the owners of the horses believed their saddle fit to be ‘ideal’, and 53% of owners had their saddles checked ‘regularly’ by a qualified professional.

By law, horse owners in the UK and many other nations are required to use only suitably qualified professionals to provide certain services necessary for horse health, i.e., veterinary [17] and farriery services [18]. However, a musculoskeletal therapist, saddle fitter, and riding coach may also provide services that directly influence the horse’s management and welfare, including matters relating to saddlery fit. Essentially, these professions form an equestrian multi-disciplinary team (MDT); however, the term and the concept of an MDT are less well established than in other sectors such as healthcare [19], farming, and sport [20]. The benefits of MDTs should be transferable to the equine industry. To date, only a limited number of studies have investigated MDT within equine healthcare; input from non-veterinary surgeons has been associated with the prevention of certain conditions, e.g., the farrier in the prevention of laminitis [21] and the veterinary physiotherapist in the prevention of pelvic and hindlimb fractures in racehorses [22]. Using qualitative methods, the experiences of inter-disciplinary workings from the perspectives of saddle fitters [23] have been investigated. Six themes of equal importance were identified: effective communication, multidisciplinary expectations, horse welfare, professionalism, relationships, and working together [23]. Fitters recognised that working with equine professionals as part of an MDT brought benefits not only for horse welfare but also for the professionals themselves, while also recognising potential barriers such as time and financial limitations [23].

MDT has been referred to as a holistic approach to problem-solving [24]. Within human healthcare, the MDT is defined as a team of healthcare professionals working together to improve care and health outcomes [19]. The ‘problem’ of suboptimal saddle fit in the horse could thus potentially be solved by a MDT approach. Since a horse’s epaxial musculature dimensions fluctuate throughout the year, influenced by seasonal changes, work quality, rider, horse body weight, and discipline [12], a saddle that is regarded as a correct fit can become out of balance in a relatively short period of time [12]. As a result, industry guidelines recommend that saddle fit be assessed biannually [25]. The frequency at which some of the members of the MDT see a horse will be greater than that of the MDT saddle fitter. Although members of the MDT may not possess a qualification in “saddle fitting”, some form of observation of saddle fit may be a necessary part of their role. This may be advantageous in early detection of incorrect saddle fit given the dynamic nature of ‘fit’, with those professions observing ridden work being well placed to recognise deterioration in saddle fit and to act upon it. However, overlap in professional roles may also lead to a failure to respect professional boundaries, something that has been reported to be a potential threat to successful MDT work. Achieving optimal saddle fit for horse and rider is dependent on various stakeholders, including the horse owner [23].

Understanding the saddle fit issues seen most frequently by all MDT members could directly impact horse welfare, comfort, and performance during ridden exercise through more effective messaging to owners. Given the potential benefits of effective MDTs and the prevalence of suboptimal saddle fit, the aims of this study were to (1) understand how professionals within an MDT interact with their clients and with others within the team on matters of saddle fit, and (2) identify what the various professions perceive as the most common issues in saddle fit for the horse and rider.

## 2. Materials and Methods

### 2.1. Participants

Participants (*n* = 483) were recruited online by sharing the survey link with UK industry regulatory bodies (Association of Chartered Physiotherapists in Animal Therapy, Register of Animal Musculoskeletal Therapists, British Horse Society, Society of Master Saddlers, and British Equine Veterinary Association), National Press (Horse and Hound, published online, April 2022), and social media (Facebook^®^, Twitter^®^, LinkedIn^®^, all California, USA) groups and pages. Inclusion criteria required participants to be over 18 years of age and be a current equestrian professional in one of the multidisciplinary groups targeted (Veterinarians, physiotherapists, chiropractors, massage therapists, coaches, farriers, dentists, and saddle fitters).

### 2.2. Survey Design

This study was designed as an online questionnaire (JISC online surveys, Bristol, UK) with 25 questions, which included 11 closed questions and 14 multiple-choice questions containing Likert scales, 6 ranking questions, and 9 open text questions. Open questions enabled respondents to describe common reasons they encountered for why saddles did not fit, as well as the opportunity to highlight additional factors or issues they observed relating to saddle fit. The survey was split into three major sections: (1) participant demographics; (2) saddle fit for the horse; and (3) saddle fit for the rider (Supplementary Material). Questions relating to bridle and bit fit were also included, the findings of which are reported in a separate manuscript. A draft questionnaire was pilot tested by 13 equestrian professionals to check its usability and edited to correct any errors before being launched. The survey was live for 68 days, and 80% of the responses were obtained within the first fourteen days. A priori sample size calculation (Survey Monkey™ Seattle, DC, USA) based on a population estimate of 18 k, identified a minimum of 377 responses to be representative of the targeted professional populations at the 95% confidence level, with a ±4% margin of error.

The survey contained the following sections:

- Demographic factors (Questions 1–7): the professional’s main profession, country of residence, qualifications, qualification awarding body, number of years qualified, professional memberships, number of horses seen professionally per week, and discipline/type of horses seen per week.
- Saddle fit for the horse (Questions 8–19): does the professional ask their clients when they last had their saddle fitted and the qualifications of the person who fitted the saddle? Does the professional assess the saddle as part of their professional service, and how do they assess saddle fit? Do the professionals make recommendations for referrals? Whom do they recommend, and what factors influence their recommendation? What saddle fitting issues does the professional encounter, and do they make any alterations to the client’s existing saddle set-up to improve saddle fit?
- Saddle fit for the rider (Questions 20–22): does the professional assess saddle fit for the rider, and how is this assessed? What saddle fitting issues in relation to rider fit does the professional encounter, and do they make any alterations to the client’s existing saddle set-up to improve saddle fit for the rider? Finally, respondents were asked if they made changes to equipment based on safety and whether they felt they had the necessary skills to judge saddle fit for the horse and rider (Questions 23–25).

### 2.3. Data Analysis

#### 2.3.1. Descriptive Analysis

Data were exported from JISC Online Surveys™ to Microsoft Excel™ Version 2020 (Redmond, WA, USA). Frequency analysis identified the respondent role, if they held qualifications, and how long they had been qualified in their respective professions, as well as how many horses they visited weekly. Frequency analyses also reported differences in the approaches used to assess the saddle by professions within client visits and the most frequently encountered issues (rated 1: nearly always, 2: very often, 3: often, 4: not often, 5: never). “Nearly always” was defined as ‘more than 90% of client visits, ’very often’ as 60–89%, often as 30–59%, not often as 1–29%, and never as 0%. For statistical analysis, “nearly always” and “very often” responses were grouped to reflect that an approach was applied in the majority (more than 60%) of client visits.

Data met non-parametric assumptions; therefore, a series of Kruskal–Wallis analyses identified if:

1. Differences existed across the cohort, i.e., between all respondent professions.
2. Differences existed between specific groups, i.e., saddle fitters versus therapists (veterinary physiotherapists, osteopaths, chiropractors, and massage therapists) versus equestrian coaches, for the number of horses seen per week, the number of years qualified, and their approach to saddle fit assessment. Veterinarians (*n* = 11) and farriers (*n* = 0) were excluded from this analysis due to low respondent numbers.
3. Differences existed between saddle fitters, therapists, and equestrian coaches regarding how they advised clients on saddle fit.

For factors where significant differences were found, Mann–Whitney U post-hoc tests identified how ratings differed between the groups. Median rankings for individual factors were examined to identify the direction of differences between disciplines; where median values were the same, mean rank differences obtained from post hoc tests differentiated between disciplines. The significance was set at *p* < 0.05.

#### 2.3.2. Univariable and Multivariable Analysis

Binary logistic regression modelling was used to identify if differences in practice occurred between respondents who were saddle fitters and the other professions [26]. A univariable analysis of each of the factors assessed was completed. Factors with a *p* value < 0.1 were included in the final multivariable model [27]. A total of 32 variables were taken forward to multivariable modelling. A multivariable logistic regression model was constructed using a backwards-stepwise process, with an omnibus test of model coefficients applied at each step. The Hosmer–Lemeshow goodness-of-fit test assessed the final models [28]. The predictive ability of the model was assessed using receiver operating characteristic (ROC) curve analysis [29,30]. Factors with a *p* value < 0.05 in the final multivariable model were considered significant [26].

#### 2.3.3. Inductive Content Analysis 

For open text responses, inductive content analysis using an open coding approach was applied to create emergent categories that described participants’ perspectives and their knowledge and understanding related to the process of saddle fitting [31].

## 3. Results

### 3.1. Demographics 

A total of 483 respondents completed the survey, with responses coming from Europe, Central/South America, and North America. The majority of respondents (*n* = 377) who completed the survey were based in the UK (6% margin of error at 95% CI). Factors influencing professional practice can vary by country and culture; therefore, to ensure the data represented a homogenous population, only responses from the UK Responses that were from the UK were analysed and reported forthwith.

The majority (92%; *n* = 346) held qualifications for their profession; 56% (*n* = 212) of respondents had been qualified for more than 11 years. Most (65%; *n* = 246) of the sample saw less than 20 horses per week. The respondents’ client base took part in a wide range of equestrian disciplines (dressage, show jumping, eventing, and leisure riding). A total of 184 saddle fitters completed the survey (4% margin of error at 95% CI); 87% (*n* = 160) held a saddle fitting qualification, and 13% (*n* = 24) did not. Saddle fitters were typically (i.e., the most frequent response) qualified between 11 and 20 years (28% and *n* = 52) and visited between 11 and 20 horses per week (47% and *n* = 87). A total of 77 therapists (vet physio, chiropractor, osteopath, and bodyworker) completed the survey (11% margin of error at 95% CI); all (100%) held professional qualifications. Fifty-six percent (*n* = 43) of therapists had been qualified for >5 years. Therapists typically saw between 11 and 20 horses per week (64%; *n* = 49). A total of 116 coaches completed the survey (9% margin of error at 95% CI). Ninety-four percent of coaches held a professional qualification (*n* = 109), and 87% (*n* = 101) had been qualified for >5 years. Coaches typically saw between 11 and 30 horses per week (61%; *n* = 71).

No significant difference was found in the qualified status between saddle fitters, therapists, and coaches (*p* = 0.103). However, there were significant differences in the number of years saddle fitters, therapists, and coaches had been qualified (*p* = 0.004); post hoc tests showed saddle fitters (80% > 5 years) had been qualified longer than therapists (*p* = 0.007; 56% > 5 years) and therapists had been qualified less time than coaches (*p* = 0.0004; 87% > 5 years). Cumulatively, significant differences existed between the number of horses seen per week between saddle fitters, therapists, and coaches (*p* = 0.002). Post hoc tests identified that saddle fitters saw more horses per week (73% visit > 10 horses/week) than therapists (*p* = 0.042; 64% visit > 10 horses/week), and therapists saw more horses per week than coaches (*p* = 0.001; 61% visit > 10 horses/week).

### 3.2. Saddle Fit for the Horse

**Figure 1:**
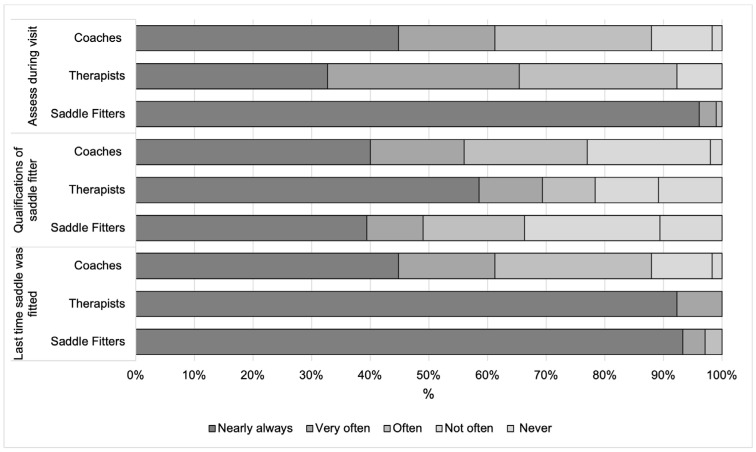
Percentage of responses from saddle fitters, therapists, and coaches when answering three questions relating to the saddle.

Most saddle fitters (97%; *n* = 178), 61% (*n* = 71) of coaches, and 100% of therapists (*n* = 77) “nearly always” or “very often” asked clients when they last had their horses’ saddle fitted, with saddle fitters (97%) and therapists (100%) asking more frequently than coaches (55%, *p* = 0.0004 for both comparisons). Forty-nine percent (*n* = 90) of saddle fitters, 55% (*n* = 64) of coaches, and (*n* = 59) 77% of therapists “nearly always” or “very often” asked clients about the qualifications of the individuals who fitted their horses’ saddles. Differences were found between saddle fitters, coaches’, and therapists’ responses when asking about the qualifications of the person who fits their client’s saddles (*p* = 0.043), with therapists asking about qualifications more than saddle fitters (*p* = 0.017). Ninety-nine percent of saddle fitters, “nearly always” or “very often”, make an assessment of saddle fit as part of their professional service, which was significantly more than coaches (61%, *n* = 82, *p* = 0.0004) or therapists (65% (*n* = 50), *p* = 0.0004). No significant difference was found between therapists and coaches at *p* ≥ 0.05 (Figure 1).

In relation to the assessment of saddle fit, saddle fitters typically subjectively assess a saddle when the horse is “standing in the stable and when ridden” (89%, *n* = 164), which differs from therapists, where it varies “depending on the circumstances of the assessment” (75%, *n* = 58, *p* = 0.0004). However, therapists were typically more likely to assess when the horse is “standing in the stable and when ridden” (27%, *n* = 21) than coaches (45%, *n* = 52), who most often assess the saddle when the horse is “standing in the stable” (*p* = 0.0004, respectively). Eighty-five percent of therapists (*n* = 65) and 87% (*n* = 101) of coaches typically recommend that their clients “use a qualified person” to check their saddle fit. No significant differences occurred in the approach taken across saddle fitters, therapists, or coaches when recommending a qualified saddle fitter (*p* > 0.05) or whether respondents recommended their clients’ use of their existing saddle (*p* > 0.05). However, when recommending clients to use a specific saddle fitter (*p* = 0.0004), saddle fitters (94%, *n* = 173) “recommend a specific saddle fitter” more than therapists (21%, *n* = 16; *p* = 0.021) and coaches (23%, *n* = 27; *p* = 0.0004).

Where saddle fitters recommend other equestrian professional services, the most influential sources are their “knowledge and experience” (32%), “qualifications” (11%), and “previous experience of working with the team” (14%). For coaches, the most influential sources were “knowledge and experience” (32%), “whether or not the coach uses them for their horse(s)” (24%), and “qualifications” (23%). For therapists, the most influential sources were “qualifications” (42%), “knowledge and experience” (38%), followed by “previous experience of working with them” and “word of mouth” (6%, respectively).

### 3.3. Saddle Fit Issues

**Figure 2:**
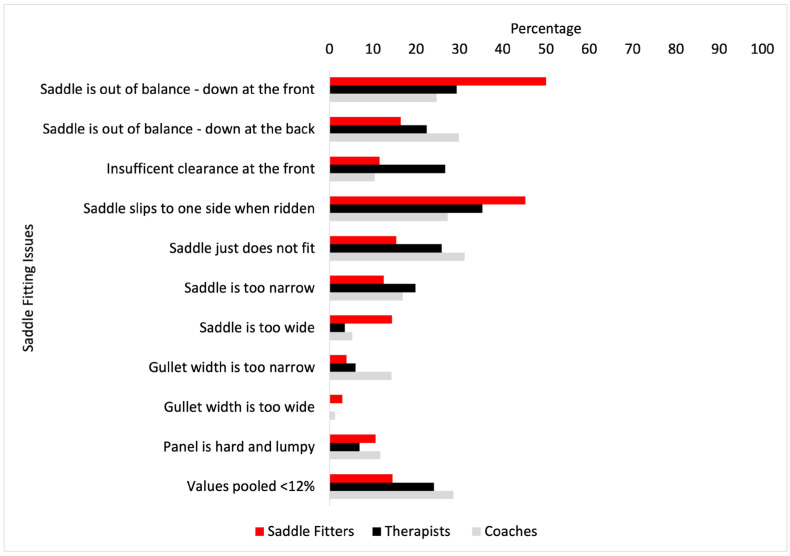
The most frequent saddle fit issues (%) encountered by saddle fitters (red bars), coaches (grey bars), and therapists (black bars). Values pooled <12% were: saddle tree is broken, tree looks crooked or twisted, saddle is too tight, not enough room for the rider, too much room for the rider, saddle is too long for the horse, and Stirrup bars are too tight.

The most frequent saddle fit issues encountered by saddle fitters, coaches, and therapists can be seen in Figure 2. Saddle fitters experienced the saddle being out of balance (down at the front) more than therapists (*p* = 0.032), but no other significant differences were found between groups.

#### Saddle Alterations

Significant differences occurred in how saddle fitters, coaches, and therapists made alterations to their clients’ existing saddle set-up. Sixty-one percent of saddle fitters “nearly always” make alterations to the saddle, 54% “very often” alter the girth or girthing arrangement, and 35% would “often” make alterations with a half pad or shims. A similar percentage (61%) of therapists would “nearly always” make alterations to the saddle, 48% would “very often” and a further 31% would “nearly always” alter the girth or girthing arrangement, and 45% would ‘very often’ and a further 27% would ‘nearly always’ make alterations with a half pad or shims. In contrast, coaches made fewer alterations; 33%, 44%, and 39% of coaches do not often (“not often”) alter the saddle, girth or girthing arrangement, half pad, or shims, respectively. Post hoc analyses found saddle fitters made more alterations to the saddle than therapists (*p* = 0.0004) and coaches (*p* = 0.0004), and therapists more than coaches (*p* = 0.0004). This pattern was repeated for “girth or girthing arrangement”, with saddle fitters changing this more than therapists (*p* = 0.0004) and coaches (*p* = 0.0004), and therapists more than coaches (*p* = 0.0004). Saddle fitters changed “half pads or shims” more than therapists (*p* = 0.0004) and coaches (*p* = 0.0004), but no differences were reported between therapists and coaches. Significant differences occurred between saddle fitters, therapists, and coaches for whether they had ever adjusted, removed, or added any piece of saddlery equipment in the interest of safety (*p* = 0.0004). Post hoc analyses found 97% of saddle fitters adjusted aspects of saddlery for safety more than therapists (42%; *p* = 0.0004). Significant differences occurred between saddle fitters, therapists, and coaches as to whether they felt confident they had the skills necessary to make judgements on saddlery fit for the horse (*p* = 0.0004). Saddle fitters (99%) were more confident than both therapists (71%; *p* = 0.0004) and coaches (68%; *p* = 0.0004) that they had the skills necessary to make judgements on the saddlery fit for the horse.

### 3.4. Saddle Fit for the Rider

The most frequent issues with saddle fit for the rider are shown in Figure 2. The most frequent issues encountered by saddle fitters were “saddle tips the rider forwards” (15%), followed by “saddle seat is too small” (14%). The most frequent issue for coaches was “saddle seat is too small” (15%), followed by “saddle slips to one side when ridden (11%). The most frequent issues for therapists were “saddle seat is too small” (27%), followed by “saddle slips to one side when ridden” (18%).

**Figure 3:**
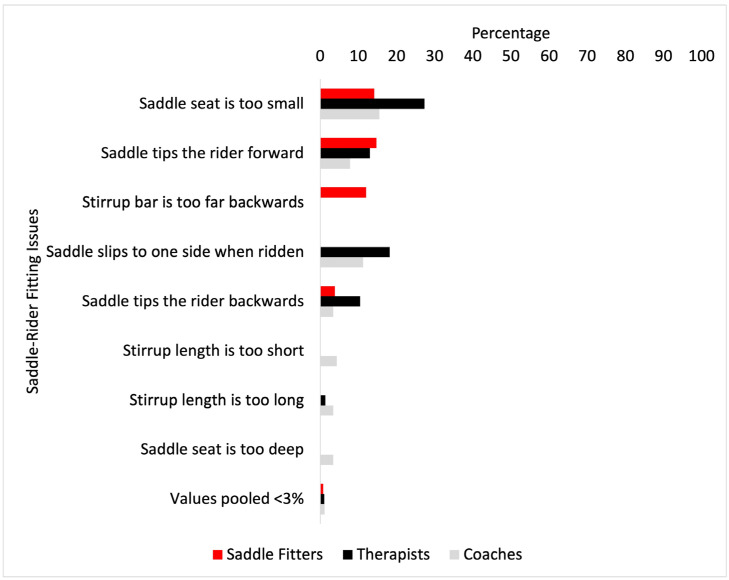
Most frequent issues of saddle fit for the rider (%) encountered by saddle fitters (red bars), coaches (grey bars), and therapists (black bars). Values pooled <3% were: stirrup size is too small for rider foot size, stirrup size is too large for rider foot size, saddle flap too small/big/wrong size and boot catches, thigh roll incorrectly positioned to influence lower leg, saddle seat is too big, knee roll is too upright—affecting the rider’s upper limb, knee roll is too small—offering no support, knee rolls are too big, knee roll restricts the rider too much, stirrup bar is too forward and the thumb grips on stirrup bar are always up.

Differences between saddle fitters, coaches, and therapists were found in the most frequent saddle fit-related issues: “saddle is too small” (*p* = <0.001), where post hoc analysis found therapists (*p* = 0.03) and coaches (*p* = 0.0001) experienced this more than saddle fitters; “saddle tips the rider forwards” (*p* = <0.001), where post hoc analysis found saddle fitters experienced this more than therapists and coaches (*p* = 0.0001, respectively); differences were found for “saddle slips to one side when ridden” (*p* = 0.01). Post hoc analysis found coaches experienced this more than saddle fitters (*p* = 0.02), and therapists experienced it more than coaches (*p* = 0.009) (Figure 3).

### 3.5. Open-Text Questions

Three themes were identified from respondents’ responses regarding recommendations for other equestrian professional services: (1) recommend using a qualified professional but not a specific individual; (2) geographic area/horse/knowledge; and (3) availability.

**Figure 4:**
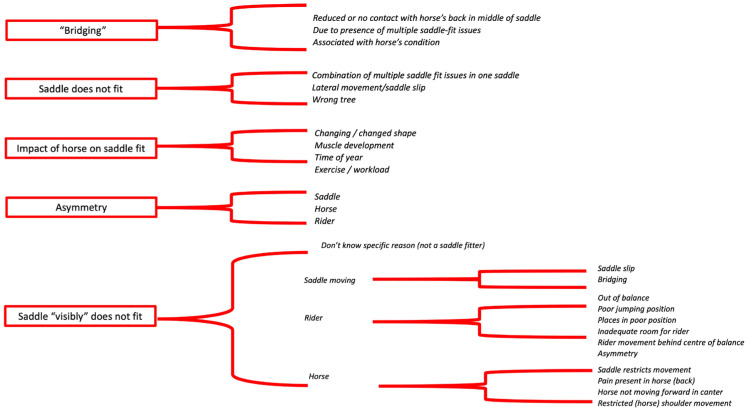
The five key themes (red boxes) and a summary of respondents’ text descriptions in relation to saddle fit issues they encountered most frequently.

Five key themes were identified from respondents’ responses regarding saddle fit issues they encountered most frequently: (1) saddle ‘bridges’, (2) the saddle just ‘does not fit’, (3) the impact of the horse on saddle fit, (4) asymmetry, and (5) the saddle ‘visibly’ does not fit (Figure 4).

**Figure 5:**
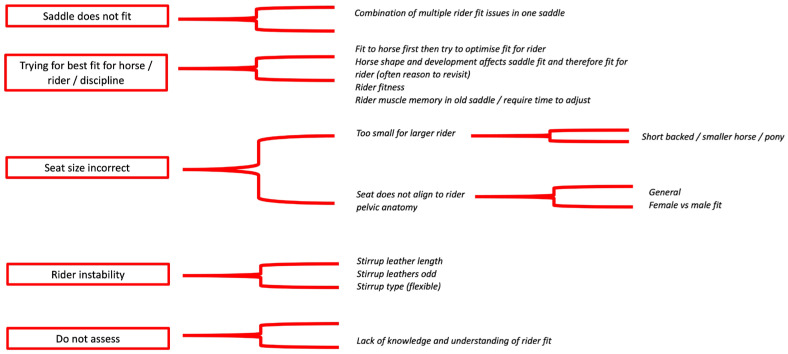
The five key themes (red boxes) and a summary of respondents’ text descriptions in relation to saddle fit for the rider issues they encountered most frequently.

Five key themes emerged across respondents for the most frequent reasons a saddle does not fit the rider: (1) saddle just does not fit; (2) trying for best fit for horse, rider, or discipline; (3) seat size incorrect; (4) rider instability; and (5) do not assess (Figure 5). 

The following variables were taken forward from univariable modelling (*p* < 0.10) to the final model:

- Number of horses seen per week; recommend a qualified saddle fitter; recommend a specific saddle fitter if they use the professional for their own horse, saddle down at the front, insufficient clearance at the front of the saddle, saddle is too wide, panel is hard/lumpy, saddle is too tight, saddle slips to one side when ridden, stirrup bars are too tight, alterations to the saddle, alterations to the girth, too much room for the rider; alterations to the half pad/shims, assess saddle-rider fit, how to assess saddle-rider fit, stirrup bar too forward, saddle fit too long, and stirrup length too long.

The following variables were retained in the final model:

- Insufficient clearance at the front of the saddle; the panel is hard and lumpy; and alterations to the half pad/shims.

Saddle fitters are significantly more likely (2.6 times) to find panels hard/lumpy more frequently than non-saddle fitters, and they are significantly more likely to often (0.2 times) and very often (0.4 times) make saddle fit alterations with a half pad or shims compared to non-saddle fitters. Finally, saddle fitters are significantly more likely to nearly always (7.6 times) and very often (28.7 times) assess saddle fit for the rider than non-saddle fitters.

## 4. Discussion 

The aims of this study were to (1) understand how professionals within an MDT interact with their clients and with others within the team on matters of saddle fit, and (2) identify what the various professions perceive as the most common issues in saddle fit for the horse and rider. The results demonstrate the interactions between saddle fitters, coaches, and therapists with their clients, providing insight into the potential for the MDT to support horseowners in achieving and maintaining optimal saddle fit. Not surprisingly, the vast majority of saddle fitters ask clients when their saddle was last fitted, with therapists enquiring to the same degree. Saddle fit is often cited as a cause of back pain [3,6], which explains why it appears to be standard practice for therapists to enquire about saddle fit. There is clearly overlap in terms of professional responsibility for saddle fit, with over 60% of coaches and therapists making an assessment of saddle fit as part of their professional role. This may ‘safeguard’ saddle fit to an extent, at least for those horseowners using these professions on a regular basis but could lead to inter-professional conflict due to a lack of clear professional boundaries [32].

The majority of saddle fitters systematically assess saddle fit both statically (i.e., in the stable) and dynamically (i.e., being ridden), fulfilling the requirements for assessment recommended by the Society of Master Saddlers. The approach that therapists take to assess saddle fit “varies depending on the circumstances of the assessment”, presumably because some of the horses they see are not fit to be ridden, and therefore the requirements for assessment of fit according to the Society of Master Saddlers cannot be met. Similarly, the fact that most coaches assess fit statically suggests that coaches interpret ‘assessment of fit’ differently from saddle fitters. To ‘assess’ means to “evaluate or estimate the nature, ability, or quality of”. To a saddle fitter, their ability to “assess’ saddle fit is part of what defines them as professionals, yet the term is not exclusive to saddle fit nor to those that have undergone formal training in saddle fitting. An improved definition of what it means to assess saddle fit and, indeed, exactly what is assessed during the process may enhance communication between professions and improve professional recognition of saddle fitters [23].

A previous study and the current study suggest that therapists may conclude that a saddle “does not fit” [20]. Given their professional role in treating musculoskeletal injury, it is likely that the therapist’s caseload includes a relatively high proportion of horses for which the saddle does not fit; however, the phrase tends not to acknowledge the transient nature of ‘fit’ and the fact that changes in the horse’s condition, muscle mass, and muscle development may be responsible for suboptimal fit. Saddle fitters have experienced conflict with other members of the MDT who have raised concerns relating to the fit of the saddle using this phrase [23]. Good communication skills are regarded by saddle fitters as pivotal to limiting any potential misinterpretations [23]. A simple change in the language used, perhaps from “the saddle does not fit” to “I think it would be useful to have your saddle fit checked”, could enhance interdisciplinary working and improve horse owner confidence in their MDT.

Saddle fitters, therapists, and coaches reported that they recommend that their clients consult with a qualified professional if a saddle-related issue is identified. In the current study, therapists and coaches did not recommend a specific saddle fitter, which may indicate that the professionals refer their clients back to their existing saddle fitter where one already exists within the team. This demonstrates a good degree of professionalism within the MDT. It was interesting that therapists, as the most highly qualified yet relatively less experienced profession, valued “qualifications” more than “knowledge and experience” when recommending any other professional service. For coaches, “knowledge and experience” was the most influential factor, which was more influential than qualifications. Encouragingly, “previous experience of working with them”, which all groups are influenced by to some extent, could be indicative of an already established MDT approach within the professional’s role. 

Descriptively, saddle fitters and coaches were more specific in the saddle fit issues that they encountered, whereas therapists selected responses that were more general in terms of the issues that they encountered. The differences in observations may reflect the professional role and the status of the horses typically seen. The most frequent saddle fitting issues across the groups were “saddle out of balance front-back”, saddle slips to one side when ridden”, and “saddle just does not fit”. No significant differences were found between groups for the most frequent saddle fit issues, with the exception that the “saddle is out of balance—down at the front”, with saddle fitters experiencing this more than therapists. Given that the majority of therapists and coaches see some form of saddle fit observation as part of their role, the high degree of overlap in the common issues seen between professions (see Figure 2) is encouraging in terms of the scope for supporting owners in improving saddle fit for the horse. While saddle-fit observations may be interpreted differently by the various professions, there is evidence from this study that similar issues are coming to light despite the differences in the lens through which they are viewed. For example, coaches claim to most often ‘assess’ saddle fit in the stable, yet still they concur with the other professions on saddle slip being one of the most frequent issues with saddle fit.

Compared to therapists and coaches, saddle fitters were more confident when making adjustments to the saddle and were significantly more likely to alter the client’s existing set-up to improve the saddle fit, which is to be expected given their role. Interestingly, 61% of therapists stated that they would “nearly always” make alterations to the saddle to improve saddle fit, despite this not being their professional area of expertise. In contrast, coaches made fewer alterations to the saddle. Given that therapists are more likely than coaches to be presented with horses with musculoskeletal health issues, they may also be more likely than coaches to witness acute changes in back musculature and posture influencing saddle fit. Direct communication between professionals in these circumstances may lead to an earlier resolution of any problems, with benefits for the horse [33].

From a rider-saddle fit perspective, the most frequent rider-saddle fit issue encountered by saddle fitters was “saddle tips the rider forwards”. This concords with the most frequent saddle fit issue, “saddle down at the front”, as reported by saddle fitters. A saddle that is down at the front is generally too wide and, as a function of saddle tree width, will cause the rider to pitch their upper body forwards [8]. Therapists and coaches experienced the same issues in that, firstly, they reported that the “saddle seat is too small”, followed by “saddle slips to one side when ridden”. A saddle that is too small for the rider will position the rider’s weight towards the cantle, which may cause areas of focal high pressure in the caudal region beneath the saddle [34]. There are several factors that potentially contribute to the relatively high incidence of the issue of ‘saddle‘ seats being too small’. Perhaps the most obvious is a mismatch between the size of the horse and the size of the rider, but the shape of the seat, the tree, the length of the rider’s thigh, and the stirrup length could all contribute to the declaration that the saddle seat is too small. Further work on what to look for in fitting saddles for riders is needed. The results of the multivariable analysis showed that saddle fitters were significantly more likely than coaches to assess saddle fit for the rider. This highlights another area where saddle fitters and coaches may work together to improve coaching outcomes and horse and rider performance. Previous studies have indicated that saddle slip can be challenging for trainers (coaches) to recognise [14]. Therefore, the findings from the current study are positive in that a saddle slipping to one side when being ridden is being recognised, which, with greater awareness of the possible causes of saddle slip (hind limb lameness [13]) and its effect on the horse [7], may prompt a proactive discussion between members of the MDT and the horse owner.

It is generally accepted that achieving optimal saddle fit for the horse is more likely if regular saddle fit assessments are undertaken. In addition to the primary benefit of improving saddle fit for the horse, greater understanding of the skill sets of others within the MDT and greater communication between professions may have secondary benefits for the professionals themselves. For example, improving saddlery fit for the rider may alleviate rider performance issues for the coach. Improving saddle fit for the horse may improve muscle development issues and/or back pain observed by the therapist. There is overlap between professions with respect to saddle fit, and a shared desire to improve saddle fit for the horse should encourage communication between professions on matters of saddle fit.

This study does have limitations; the level of rider/horse that the professionals work with was not investigated. In addition, it is acknowledged that some of the questions were more difficult for certain professions to answer than others, but this was deemed preferable to generating separate ‘streams’ and reducing the number of responses to each question. Some of the multiple-choice questions and open-text responses were subject to overlap. Such as in all multi-choice survey-based studies, respondents are motivated to participate and may not represent the general view. While reflective of the UK saddlery industry, the terminology and wording of some of the questions were subjective and may not have been familiar to the professionals taking part in the survey. Furthermore, this study only presents data from UK responses. It is appreciated that these findings may not translate to MDTs outside of the UK. Future work should attempt to investigate professionals working in relation to saddle fit from other countries.

If this study were to be repeated, defining words that are relative to the individual’s profession may be useful. For example, recognising that only ‘fitters’ would be trained to fully evaluate saddle fit, the term ‘observation of saddle fit’ may be more appropriate for professions not primarily trained in fitting. This study has provided evidence that there are professionals other than saddle fitters who deem saddle fit assessment to be part of their role and are prepared to be proactive in supporting the owner in achieving optimal saddle fit. These professionals could perhaps be better supported via education, either through their initial training or as part of their continuing professional education. Increased vigilance from all professions with a responsibility for horse welfare would be a positive step towards improving the standard of saddlery fit for the horse.

## 5. Conclusions

This study investigated how equestrian professionals assess, manage, and act upon saddle fit. This survey found encouraging signs of successful MDT workings and insights that stakeholders can explore to promote and ensure a sustainable and cohesive MDT approach. Compared to coaches, saddle fitters and therapists asked more frequently when their clients last had their saddle fitted. In accordance with industry guidelines, saddle fitters assessed the saddle statically and dynamically compared to therapists. Unsurprisingly, saddle fitters experienced the saddle being out of balance more than therapists and made more alterations to the saddle than therapists and coaches. The survey found similar saddle fit issues were frequently identified by all professions taking part with regard to saddle fit, not only for the horse but also for the rider, despite inter-disciplinary differences in practice. These findings are encouraging with regard to earlier detection of saddle fit issues by horse-owners who engage regularly with equestrian professionals working as a multi-disciplinary team.

## Data Availability

Anonymised data are available on request.

## Supplementary Materials

The following supporting information can be downloaded at: https://www.mdpi.com/article/10.3390/ani14172495/s1. A full survey is provided.
