# Supplementary material for: The Role of Equestrian Professionals in Saddle Fit for Horses and Riders in the United Kingdom
Source: Animals (Basel). 2024 Aug 28;14(17):2495. doi: 10.3390/ani14172495 (PMC11394139; doi:10.3390/ani14172495)
Supplement: Supplementary file 1 [file animals-14-02495-s001.zip › animals-3146291-supplementary.pdf]

Equestrian Professionals and Saddle Fitv5

Page 1: Declaration of consent

This survey takes approximately 10 minutes to complete. All responses are anonymous. No personal data allowing identification of a respondent is required. By participating in this survey, you are agreeing to the inclusion of your responses in a research project approved by Hartpury University Research Ethics Committee number Number 2021-x Please confirm your consent by ticking 'I agree' below

- I agree
- I don't agree

Page 2: Section 1: You and Your Profession

What is your main profession? ✱ *Required*

In which country do you reside?

Please list your professional memberships, e.g. BEVA, UKCC, RAMP etc.

If you are a saddle fitter, what is your affiliation?

How long have you been qualified in your main profession? ✱ *Required*

- 0-3 years
- 4-5 years
- 6-10 years
- 11-20 years
- 21+ years
- I am not qualified

In your main professional capacity, how many horses do you see per week? ✱

Required

- (selected) Less than 10
- 11-20
- 21-30
- More than 31

In your main professional capacity, what are the top 3 disciplines/types of horses and riders you work with most frequently? (with 1 being most frequent) ✱ *Required*

	1 = most frequent	2	3
Dressage	☐	☐	☐
Show jumping	☐	☐	☐
Event	☐	☐	☐
Hunting	☐	☐	☐
Driving	☐	☐	☐
Recreational riders	☐	☐	☐
Pony Club/Riding Club	☐	☐	☐
Showing	☐	☐	☐
Racing	☐	☐	☐
Polo	☐	☐	☐
Endurance	☐	☐	☐
Western	☐	☐	☐
Other	☐	☐	☐

Page 3: Section 2a: Saddle, Bridle, and Bit Fit for the Horse

Within this section, the frequencies are defined as follows for the number of horses seen per week:

Nearly Always = more than 90%

Very Often = 60-89%

Often = 30-59%

Not Often = 1-29%

Never = 0%

Do you ask your clients when they last had their saddle, bridle, or bit fitted?

	✱ *Required*				
	Nearly always	Very often	Often	Not often	Never
Saddle	○	○	○	○	○
Bridle	○	○	○	○	○
Bit	○	○	○	○	○

Do you ask your clients about the qualifications of the person who fitted their saddle, bridle, or bit?

	✱ *Required*				
	Nearly always	Very often	Often	Not often	Never
Saddle Fit	○	○	○	○	○
Bridle Fit	○	○	○	○	○
Bit Fit	○	○	○	○	○

Do you make an assessment of saddle, bridle, or bit fit as part of your professional service?

	✱ *Required*				
	Nearly always	Very often	Often	Not often	Never
Saddle Fit	○	○	○	○	○
Bridle Fit	○	○	○	○	○
Bit Fit	○	○	○	○	○

How do you assess saddle, bridle, and bit fit?

	✱ *Required*				
	I always assess when the horse is standing in the stable	I always assess when ridden	I always assess both in the stable and ridden	It varies, depending on the purpose of my assessment	I never assess fit
Saddle Fit	○	○	○	○	○
Bridle Fit	○	○	○	○	○
Bit Fit	○	○	○	○	○

If you recommend your client has their saddle, bridle, or bit checked, which of the following apply?

	Saddle Fit	Bridle Fit	Bit Fit
I recommend that my clients use a qualified person	☐	☐	☐
I recommend that my clients contact their existing fitter	☐	☐	☐
I recommend a specific individual	☐	☐	☐
I do not make specific recommendations	☐	☐	☐
None of these apply	☐	☐	☐

If you recommend ANY other equestrian professional service, what top 3 factors influence your recommendation? (with 1 being most influential)

	1 = most influential	2	3
Whether or not I use them myself for my own horse/s	☐	☐	☐
Their knowledge and experience	☐	☐	☐
Their qualifications	☐	☐	☐
My client's feedback	☐	☐	☐
Word of mouth recommendation from other professionals	☐	☐	☐
Previous experience of working with them	☐	☐	☐
My personal relationship with the professional	☐	☐	☐
Other	☐	☐	☐

If 'other' please give details

Page 4: Section 2b: Saddle Fit Issues

From the following list, please identify the top 3 saddle fit issues you encounter in order of frequency (1 being most frequent)

	1 = most frequent	2	3
Saddle is out of balance – down at the front	☐	☐	☐
Saddle is out of balance – down at the back	☐	☐	☐
Insufficient clearance at the front	☐	☐	☐
Saddle is too narrow	☐	☐	☐
Saddle is too wide	☐	☐	☐
Gullet width is too narrow	☐	☐	☐
Gullet width is too wide	☐	☐	☐
Panel is hard and lumpy	☐	☐	☐
Saddle just does not fit	☐	☐	☐
Saddle tree is broken	☐	☐	☐
Tree looks crooked or twisted	☐	☐	☐
Saddle is too tight	☐	☐	☐
Saddle slips to one side when ridden	☐	☐	☐
Not enough room for the rider	☐	☐	☐
Too much room for the rider	☐	☐	☐
Saddle is too long for the horse	☐	☐	☐
Stirrup bars are too tight	☐	☐	☐

Other - please comment

Do you ever make alterations to your clients existing saddle set up to improve saddle fit?

	✱ *Required*				
	Nearly always	Very often	Often	Not often	Never
Saddle	○	○	○	○	○
Girth or girthing arrangement	○	○	○	○	○
Half pad or shims	○	○	○	○	○

Page 5: Section 2c: Bridle Fit Issues

From the following list, please identify the top 3 bridle fit issues you encounter most frequently (with 1 being most frequent) ✱ *Required*

	1 = most frequent	2	3
Headpiece design – shape in relation to anatomy	☐	☐	☐
Headpiece design – anatomical	☐	☐	☐
Browband – too small	☐	☐	☐
Browband – too big	☐	☐	☐
Browband attachment to the headpiece in relation to anatomy	☐	☐	☐
Noseband design – nose part	☐	☐	☐
Noseband design – head part	☐	☐	☐
Noseband width	☐	☐	☐
Noseband height in relation to head anatomy – too high	☐	☐	☐
Noseband padding – not enough	☐	☐	☐
Noseband padding – too much	☐	☐	☐
Noseband height in relation to head anatomy – too low	☐	☐	☐
Asymmetrical noseband position (twisting) during exercise	☐	☐	☐
Buckle height in relation to anatomy	☐	☐	☐
Noseband too tight	☐	☐	☐
Reins too long	☐	☐	☐
Other	☐	☐	☐

Other - please comment

Do you ever remove, add or alter bridle parts to improve bridle fit?

	✱ *Required*			
	Always	Sometimes	Rarely	Never
Headpiece	○	○	○	○
Browband	○	○	○	○
Noseband	○	○	○	○
Cheek pieces	○	○	○	○
Noseband pieces	○	○	○	○
Reins	○	○	○	○

Page 6: Section 2c: Bit Fit Issues

From the following list, please identify the top 3 bit fit issues you encounter most frequently (with 1 being most frequent)

	1 = most frequent	2	3
Bit too small	☐	☐	☐
Bit too big	☐	☐	☐
Bit is too thick	☐	☐	☐
Bit it too narrow	☐	☐	☐
Wrong bit shape for the horse’s mouth	☐	☐	☐
Bit is not the most suitable for horse	☐	☐	☐
Bit is not the most suitable for horse-rider combination	☐	☐	☐
Other	☐	☐	☐

If other - please comment

Do you ever change a bit or alter a client's bridle to improve bit fit? ✱ *Required*

- ◯ Often
- ◯ Sometimes
- ◯ Rarely
- ◯ Never

Page 7: Section 3: Saddle Fit for the Rider

Do you assess the fit of the saddle for the rider? ✱ *Required*

- Often
- Sometimes
- Rarely
- Never

How do you assess saddle fit for the rider?

- I don’t
- Mounted but stationary
- I try to observe walk, trot and canter on both left and right rein
- Always walk, trot, canter on both reins and jump (if appropriate)

From the following list, please identify the top 3 issues you encounter regarding saddle fit for riders (with 1 being the most frequent) *Optional*

	1 = most frequent	2	3
Saddle seat is too small	☐	☐	☐
Saddle seat is too big	☐	☐	☐
Saddle seat is too deep	☐	☐	☐
Saddle tips the rider forwards	☐	☐	☐
Saddle tips the rider backwards	☐	☐	☐
Knee roll is too upright – affecting the rider’s upper limb	☐	☐	☐
Knee roll is too small – offering no support	☐	☐	☐

Knee rolls are too big	☐	☐	☐
Knee roll restricts the rider too much	☐	☐	☐
Stirrup bar is too forward	☐	☐	☐
Stirrup bar is too far backwards	☐	☐	☐
Saddle slips to one side when ridden	☐	☐	☐
Thumb grips on stirrup bar are always up	☐	☐	☐
Saddle is too long for the horse	☐	☐	☐
Stirrup length is too short	☐	☐	☐
Stirrup length is too long	☐	☐	☐
Stirrup size is too small for rider foot size	☐	☐	☐
Stirrup size is too large for rider foot size	☐	☐	☐
Flap too small/big/wrong size and boot catches	☐	☐	☐
Thigh roll incorrectly positioned to influence lower leg	☐	☐	☐
Other	☐	☐	☐

If other please comment

Have you ever adjusted, removed or added any piece of saddlery equipment in your professional role in the interests of safety?

- ○ Yes
- ○ No

Do you feel confident that you have the skills necessary to make judgements on saddlery fit for the horse?

- Yes
- No
- Not sure

Do you feel confident that you have the skills necessary to make judgements on saddlery fit for the rider?

- Yes
- No
- Not sure

Page 8: Final page

Thank you so much for completing our survey!

Key for selection options

2 - What is your main profession?

- Veterinarian
- Veterinary physiotherapist
- Farrier
- Osteopath
- Chiropractor
- Coach or instructor
- Body worker or massage therapist
- Saddle fitter
- Other
